# A continuous visualization workflow for endoscopic papillectomy using balloon-assisted endoscopic ultrasound and direct cholangioscopy

**DOI:** 10.1055/a-2924-5639

**Published:** 2026-08-25

**Authors:** Xiao Hu, Shan-Shan Hu, Xiao-Gang Liu, Wei-Hui Liu

**Affiliations:** 1 Department of Gastroenterology and Hepatology 89669 Sichuan Provincial People's Hospital, School of Medicine, University of Electronic Science and Technology of ChinaChengduSichuan ProvinceChina

Endoscopic papillectomy (EP) for ampullary adenoma is limited by two major visualization challenges: unreliable preprocedural endoscopic ultrasound (EUS) staging caused by luminal instability and inability to directly assess intraductal residual disease after resection. 1​ 2​ 3​ ​4​ We report a continuous visualization workflow combining balloon-assisted EUS and direct cholangioscopy for comprehensive assessment during EP 5​ ( Video 1 ).

**Video 1** A continuous visualization workflow for endoscopic papillectomy using balloon-assisted EUS and direct cholangioscopy. Video URI: .


**Fig. 1:**
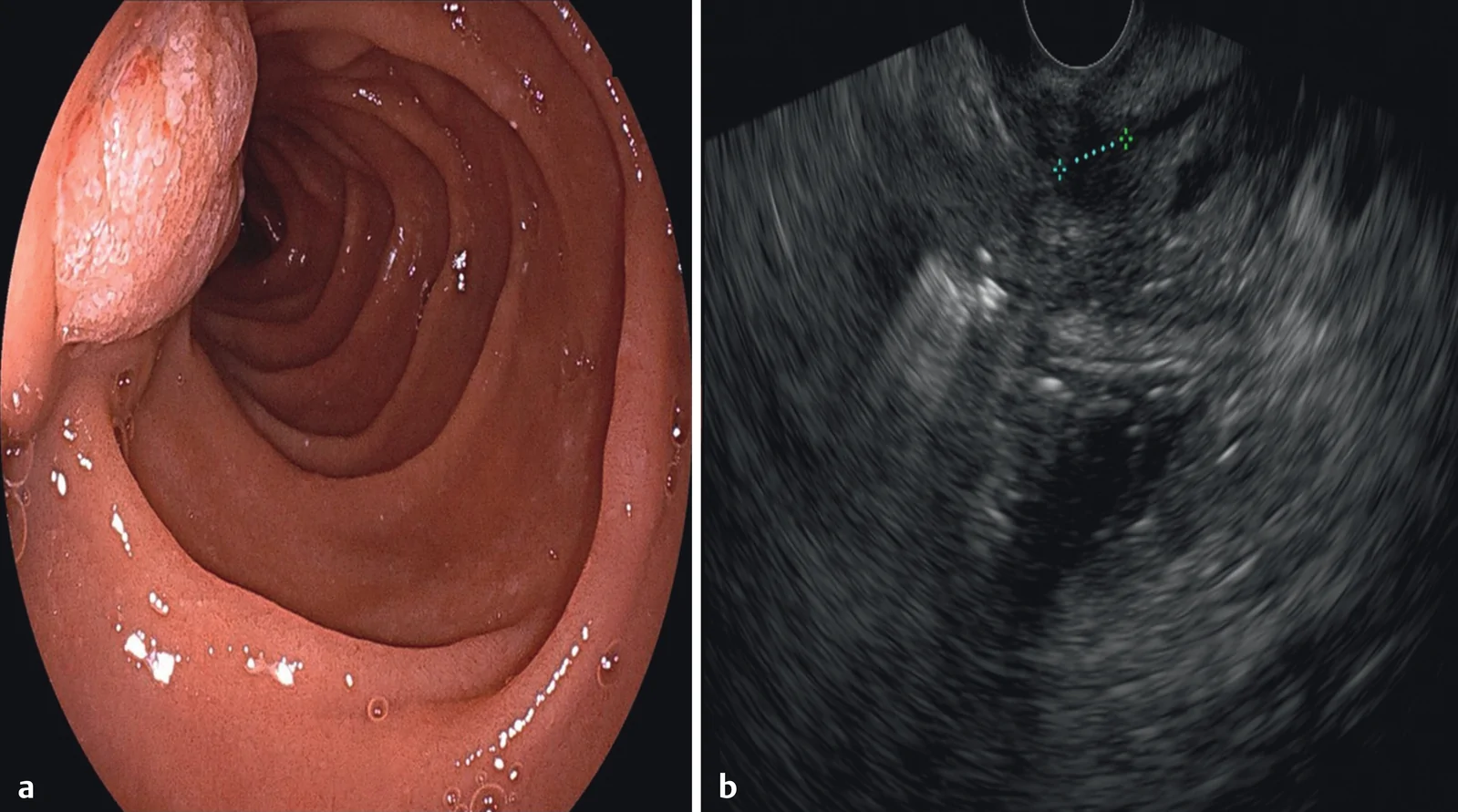
Ampullary adenoma ( **a** ) and conventional water-immersion EUS before balloon occlusion ( **b** ).

A 55-year-old man presented with abdominal discomfort for 2 months. Abdominal computed tomography revealed a 12-mm mass at the major duodenal papilla. Endoscopic biopsy confirmed ampullary adenoma with low-grade dysplasia. He was referred for EP after multidisciplinary discussion. Initial water-immersion EUS showed poorly visualized distal biliopancreatic ductal termini because of luminal collapse and rapid fluid outflow, making ductal extension difficult to exclude ( Fig. 1 ).

**Fig. 2:**
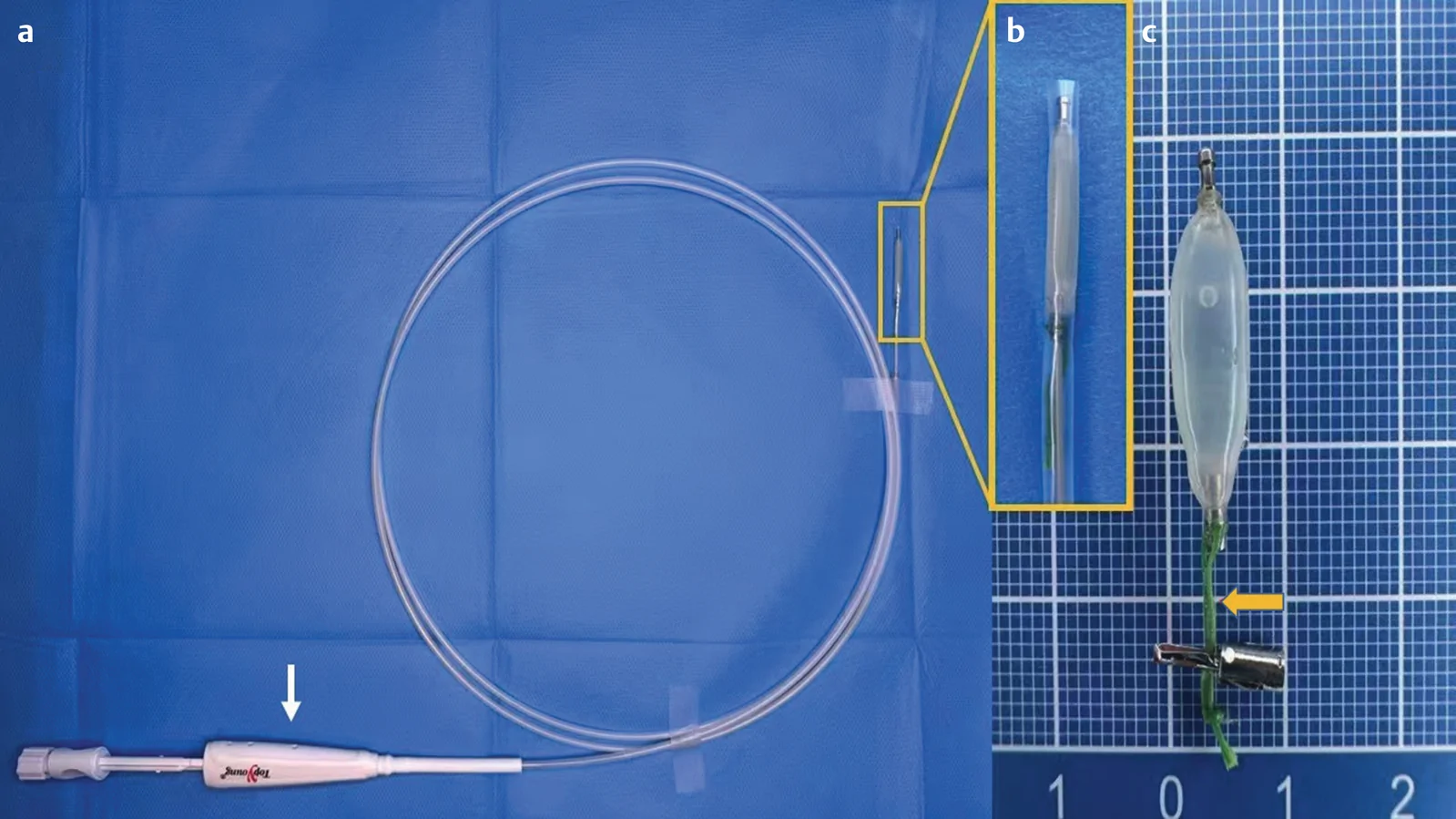
The detachable balloon occlusion device consists of three components: ( **a** ) a deployment handle (shown by the white arrow), ( **b** ) a detachable balloon (highlighted in yellow), and ( **c** ) a traction line (shown by the yellow arrow).

A detachable balloon occlusion device was deployed distal to the papilla, inflated with 20 mL saline, and secured with a clip, enabling stable luminal distension ( **Figs.** 2 **and** 3 ). Repeat EUS after balloon occlusion provided sustained visualization of the distal bile duct and pancreatic duct termini, demonstrating a lesion confined to the mucosal layer without ductal invasion ( Fig. 4 ), thereby supporting the feasibility of EP.

**Fig. 3:**
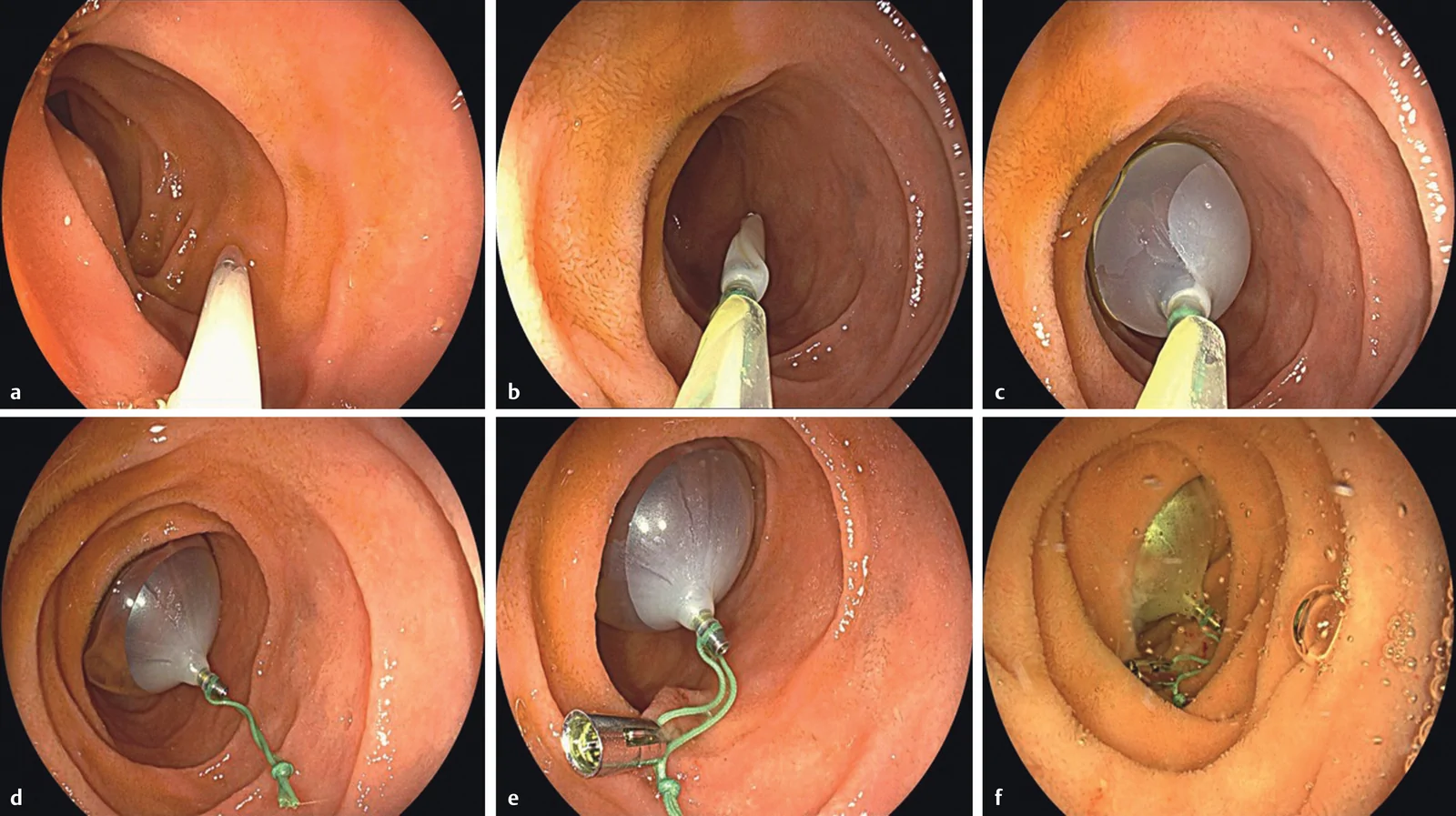
Deployment of the detachable balloon occlusion device. ( **a** ) Advancement of the balloon catheter distal to the papilla. ( **b** ) Balloon deployment in the descending duodenum. ( **c** ) Balloon inflation with saline to achieve luminal occlusion. ( **d** ) Stable luminal distension after balloon inflation. ( **e** ) Clip fixation of the traction line. ( **f** ) Endoscopic appearance after successful balloon deployment.

**Fig. 4:**
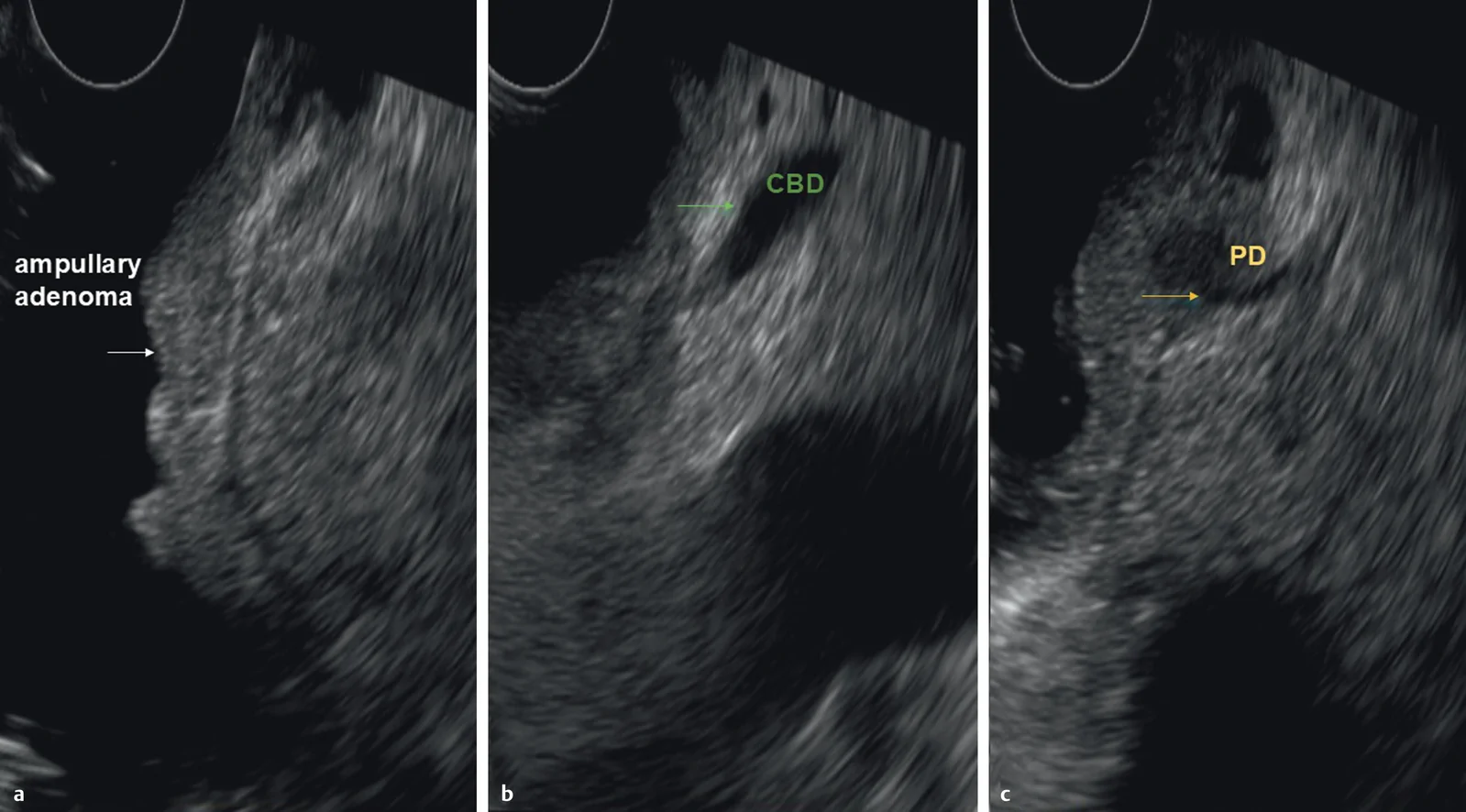
Balloon-assisted water-immersion EUS after luminal stabilization. ( **a** ) The lesion confined to the mucosal layer without ductal invasion. ( **b** ) The distal bile duct termini were clearly visualized. ( **c** ) The distal pancreatic duct termini were clearly visualized.

**Fig. 5:**
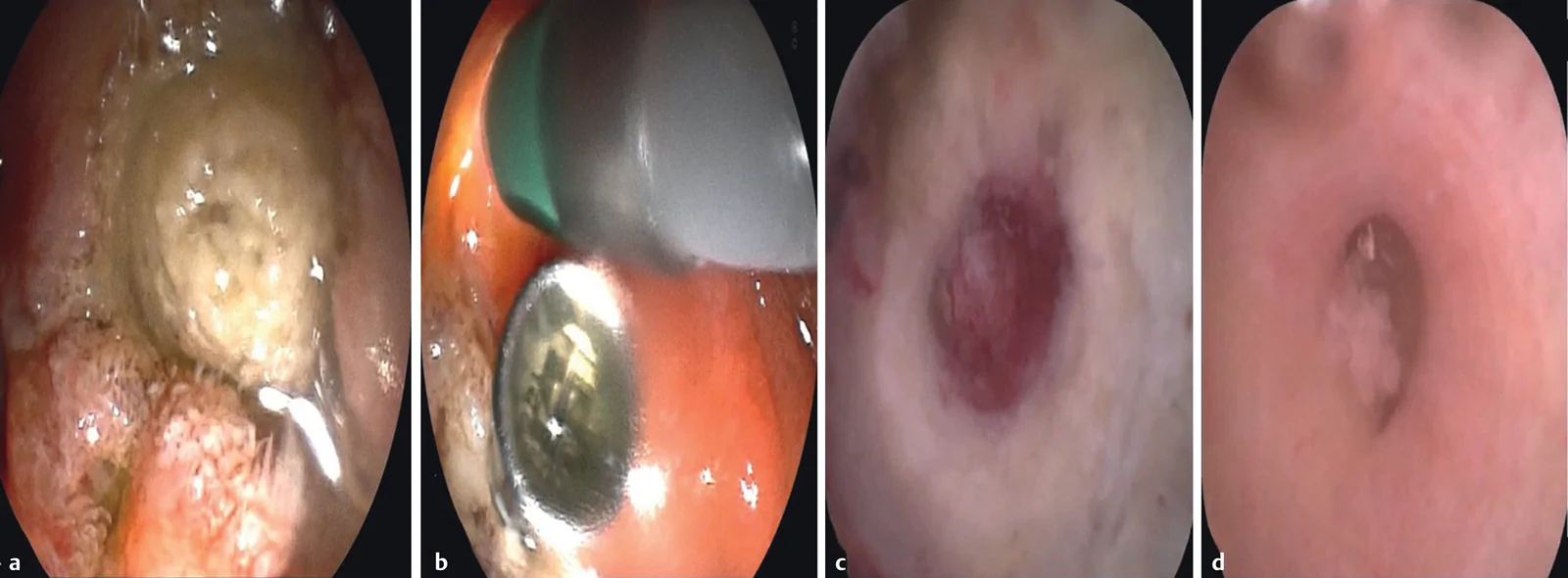
Post-papillectomy intraductal assessment using cone-cap-assisted direct cholangioscopy. ( **a** ) An endoscopic view of the postresection area before the intraductal assessment. ( **b** ) Cone-cap-assisted direct cholangioscopy. ( **c** ) Direct visualization of the distal bile duct without the residual adenomatous tissue. ( **d** ) Direct visualization of the pancreatic duct without the residual lesion.

Following en bloc snare papillectomy, cone-cap-assisted direct cholangioscopy was performed. Direct intraductal visualization confirmed the absence of residual adenomatous tissue within the distal bile duct and pancreatic duct ( Fig. 5 ). No intraoperative perforation or bleeding occurred. The patient was monitored for 48 hours after the procedure, and no clinically significant pancreatitis, bleeding, or perforation developed. He was discharged on day 3. Histopathological examination confirmed an adenoma with low-grade dysplasia and an R0 resection. Follow-up imaging during 6 to 12 months revealed no evidence of recurrence.

This workflow addresses two major visualization limitations during EP by reducing EUS staging uncertainty and enabling direct postresection intraductal assessment. The combination of balloon-assisted EUS and direct cholangioscopy may help improve procedural confidence and support accurate treatment selection for ampullary lesions.

Endoscopy_UCTN_Code_TTT_1AR-2AD
